# A Resonant Z-Axis Aluminum Nitride Thin-Film Piezoelectric MEMS Accelerometer

**DOI:** 10.3390/mi10090589

**Published:** 2019-09-06

**Authors:** Jian Yang, Meng Zhang, Yurong He, Yan Su, Guowei Han, Chaowei Si, Jin Ning, Fuhua Yang, Xiaodong Wang

**Affiliations:** 1 Engineering Research Center for Semiconductor Integrated Technology, Institute of Semiconductors, Chinese Academy of Sciences, Beijing 100083, China; 2 Center of Materials Science and Optoelectronics Engineering, University of Chinese Academy of Sciences, Beijing 100049, China; 3 State Key Laboratory of Transducer Technology, Chinese Academy of Sciences, Beijing 100083, China; 4 Beijing Academy of Quantum Information Science, Beijing 100083, China; 5 School of microelectronics, University of Chinese Academy of Sciences, Beijing 100049, China; 6 Beijing Engineering Research Center of Semiconductor Micro-Nano Integrated Technology, Beijing 100083, China

**Keywords:** MEMS, AlN thin film, piezoelectric effect, resonant accelerometer, z-axis

## Abstract

In this paper, we report a novel aluminum nitride (AlN) thin-film piezoelectric resonant accelerometer. Different from the ordinary MEMS (micro-electro-mechanical systems) resonant accelerometers, the entire structure of the accelerometer, including the mass and the springs, is excited to resonate in-plane, and the resonance frequency is sensitive to the out-plane acceleration. The structure is centrosymmetrical with serpentine electrodes laid on supporting beams for driving and sensing. The stiffness of the supporting beams changes when an out-plane inertial force is applied on the structure. Therefore, the resonance frequency of the accelerometer will also change under the inertial force. The working principle is analyzed and the properties are simulated in the paper. The proposed AlN accelerometer is fabricated by the MEMS technology, and the structure is released by an ICP isotropic etching. The resonance frequency is 24.66 kHz at a static state. The quality factor is 1868. The relative sensitivity of this accelerometer, defined as the shift in the resonance frequency per gravity unit (1 g = 9.8 m/s^2^) is 346 ppm/g. The linearity of the accelerometer is 0.9988. The temperature coefficient of frequency (TCF) of this accelerometer is −2.628 Hz/°C (i.e., −106 ppm/°C), tested from −40 °C to 85 °C.

## 1. Introduction

MEMS accelerometers and gyroscopes are widely used in inertial navigation systems [1]. The MEMS accelerometers can be categorized into electrostatic types, piezoelectric, piezoresistive, and pyroelectric ones, based on the working principles [2,3,4,5,6,7,8]. Compared with the electrostatic accelerometers (such as the Si accelerometers), the piezoelectric ones have a higher energy transfer efficiency and a simpler structure. In the electrostatic types, a complicated comb structure is a common design, since a capacitor structure is needed for excitation or sensing. The situation tends to be convenient when it comes to the piezoelectric ones, for even a simple electrode could realize the excitation or sensing functions [9,10]. Due to the charge leak, the resonant structure is often used for piezoelectric accelerometers.

**Figure 1:**
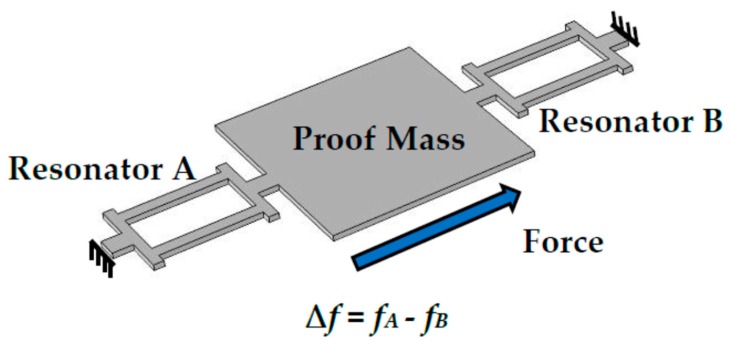
The structure of the resonant micro-electro-mechanical systems (MEMS) accelerometer.

A typical MEMS resonant accelerometer contains one or several resonant elements and proof masses, as shown in Figure 1 [7]. The resonant elements as a sensing part work in the resonant state. When an in-plane inertial force is applied on the MEMS structure, the proof mass will tense or compress the resonant elements. This will lead to a change of the stiffness. Therefore, the resonance frequency will shift with the increase of the in-plane inertial accelerations.

AlN MEMS accelerometers have been studied in recent years [11,12,13,14]. However, most of these AlN accelerometers can only detect the in-plane acceleration. This limits the integration of the AlN 3D accelerometer. To detect the out-plane acceleration, a T-shape AlN MEMS accelerometer was designed in my previous work [15]. This accelerometer resonates in-plane but measures the acceleration out-plane. The z-axis sensitivity is 68.9 ppm/g and the quality factor is 1464. In order to improve the sensitivity and Q value, an optimized AlN accelerometer is proposed in this work. 

In this paper, a tuning-fork structure is used in this AlN accelerometer. The tuning-fork structure comes from a Si MEMS gyroscope, in the Georgia Institute of Technology [16]. Two proof masses resonate reversely. In addition, the two anchors are designed at the middle of the supporting beams. When resonating, there will be no stress and no displacement at the anchors. Therefore, the anchor loss is zero. This design will benefit for improving the quality factor. This accelerometer is excited in-plane but can measure the out-plane acceleration. When an out-plane inertial force is applied on the AlN thin-film structure, the stiffness of the supporting beams will change. Therefore, the resonance frequency will shift with the increase of the out-plane acceleration. In this work, the z-axis sensitivity is measured at 346 ppm/g with the base frequency of 24.66 kHz. The quality factor is 1868.

## 2. Design

**Figure 2:**
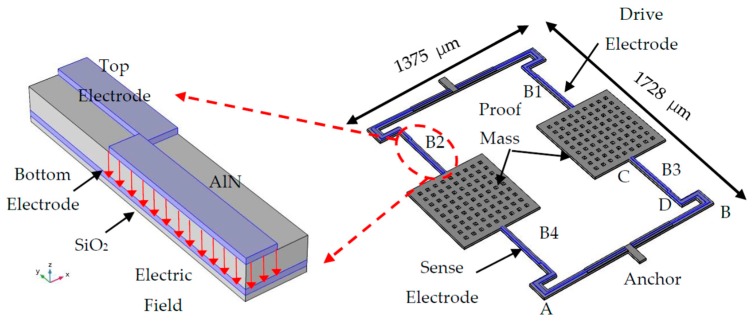
The structure of the aluminum nitride (AlN) MEMS accelerometer. In addition, the detailed analysis of the core beams (B1, B2, B3, B4).

The AlN MEMS accelerometer is composed of four supported beams and two proof masses. The schematic structure is as shown in Figure 2. It contains four layers, the SiO_2_ layer, the bottom electrode layer (Mo), the AlN layer, and the top electrode layer (Ti/Pt). The parameters are listed in Table 1. The SiO_2_ layer works as the support and insulation. The bottom electrode is connected to the ground. The top electrodes, which are serpentine, are placed on the supported beams. They contain the drive electrode and sense electrode. The drive signal is applied on the drive electrode. Due to the piezoelectric effect, the electric field will excite a mechanical strain. This strain will lead to a stress σ, which is the in-plane direction. Moreover, the stress can drive the structure to vibrate.

(1) $$\sigma =E_{ALN}\frac{U}{d_{AlN}}d_{31}$$

where *E_AlN_* is the Young’s modulus of AlN. *d_AlN_* is the thickness of the AlN film. *U* is the drive voltage. *d*_31_ is the piezoelectric coefficient of AlN. According to the reported references [17,18], the *d*_31_ = −2.6 pm/V.

**Figure 3:**
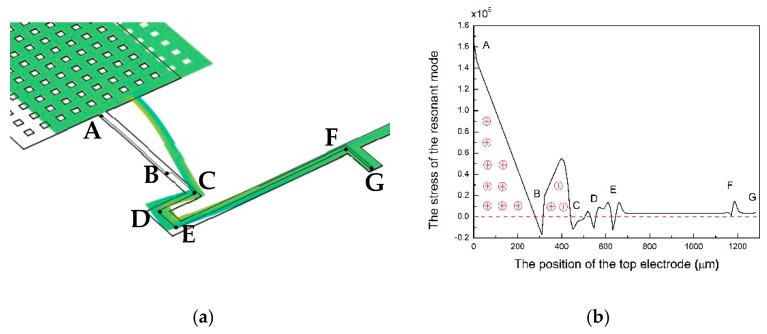
The stress simulation of the AlN MEMS accelerometer (**a**); the stress distribution along the top electrode (**b**).

To maximize the electromechanical signal, the top electrodes on the core beams are designed as serpentine electrodes. The sizes and the position of the serpentine electrodes come from the stress simulation. Figure 3a shows the stress simulation. The top electrodes are designed according to the stress distribution [11]. ABCDEFG is the line of the top electrode. Figure 3b shows the stress distribution. There are nearly no opposite sign charges on the top electrode. Therefore, a maximized electromechanical signal can be collected by the serpentine electrodes. Table 2 lists the parameters of each material. 

**Figure 4:**
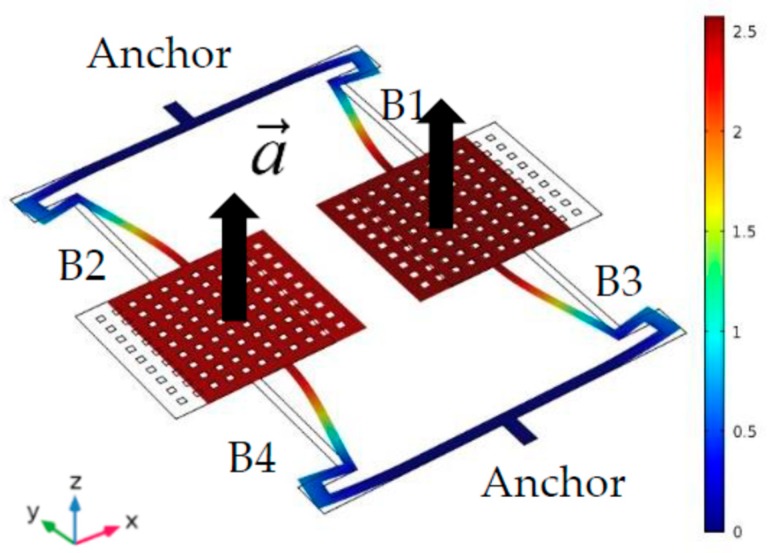
The displacement resonant mode of this AlN MEMS accelerometer.

Due to the AlN piezoelectric effect, the MEMS structure is excited to resonate in-plane by the drive signal [19]. The resonant mode is simulated by the software COMSOL Multiphysics. The simulation result is shown in Figure 4. The two proof masses vibrate reversely. The positions of the anchors are the nodes. There is no displacement and no stress. This means there will be no anchor loss. The quality factor of this accelerometer will increase [20]. 

The proof masses vibrate along the x-axis. The acceleration is along the z-axis. When an acceleration $\vec{a}$ applies on the entire structure, the supporting beams will deform in the z-axis. The stiffness coefficient *k_z_* will change. As a result, the resonance frequency *f_x_* will shift with the z-axis acceleration. The detailed derivation is in reference [15].

(2) $$f_{x}=f_{x0}\left(1-\frac{1}{2}\frac{\Delta k_{x}}{k_{x0}}\right)=f_{x0}\left(1-\frac{3a}{E_{z}}\frac{Lm}{wt^{3}}a\right)=f_{x0}\left(1-C\frac{Lm}{wt^{3}}a\right)$$

where *f*_x0_ is the in-plane resonance frequency at static state. And *k*_x0_ is the stiffness coefficient along x-axis at static state. *C* is a constant. L is the length of the beam. w is the width of the beam. t is the thickness of the structure.

The sensitivity of this accelerometer is proportional to $\frac{Lm}{wt^{3}}$. In order to increase the sensitivity, the mass and the length of the beam should be increased. In this work, the AlN MEMS accelerometer is a thin-film structure. The whole thickness of the structure is 2.05 μm. The size of the proof mass is increased to 500 μm × 500 μm.

**Figure 5:**
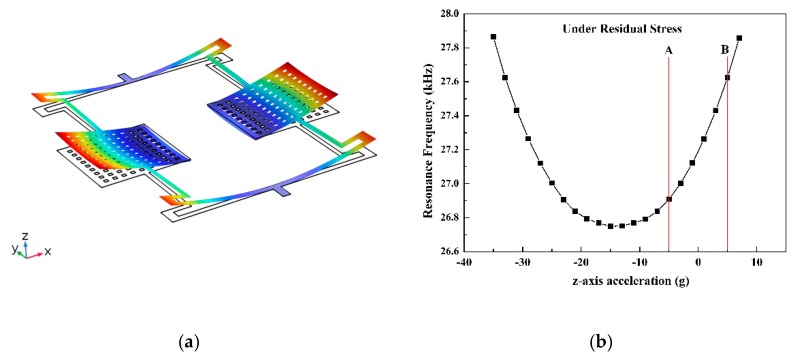
The deformation of the structure under the residual stress (**a**); the sensitivity curve of this AlN MEMS accelerometer simulated with the residual stress (**b**).

Due to the lattice mismatch, there will be a residual stress in the actual accelerometer structure. The mean value of the residual stress is more than 500 MPa. This residual stress will lead the MEMS structure to bend out of plane, as shown in Figure 5a. To simplify the simulation and show the trend of the whole curve, the residual stress is set to 5 MPa, which is less than the actual value. Figure 5b shows the simulation result with the residual stress. The AB part which includes zero g, is monotonically increasing. This part is the range of this accelerometer. 

## 3. Fabrication

The substrate is the (100) silicon wafer with a 300 nm SiO_2_ layer. The bottom electrode is Mo, with 300 nm thick. The (002) oriented AlN film is deposited on the Mo layer by the magnetron sputtering process. It is 1.3 μm thick. The piezoelectric coefficient d_33_ of the AlN film is 5.09 pm/V tested by the piezoresponse force microscopy [21]. A composite top electrode layer 150 nm Ti/Pt is deposited by the electron beam evaporation. The normal MEMS processes have been used to fabricate this accelerometer. The AlN film is etched by the ICP process with Cl_2_/BCl_3_/Ar [22]. The entire structure is released by the ICP isotropic etching process with SF_6_.

**Figure 6:**
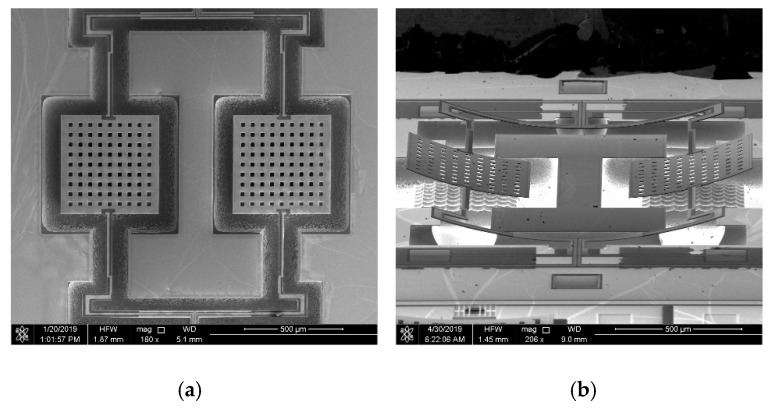
The micrographs of the AlN MEMS accelerometer. (**a**) Top view; (**b**) side view.

Figure 6a,b are the scanning electron microscope (SEM) pictures of the fabricated accelerometer. Figure 6a is the top view of the structure. Figure 6b is the side view of the structure. The structure bends along the +z-axis because of the residual stress.

In order to achieve a better performance, a vacuum package process—the discharge welding—was used. In this process, the vacuum is 0.16 Torr.

## 4. Results and Discussion

**Figure 7:**
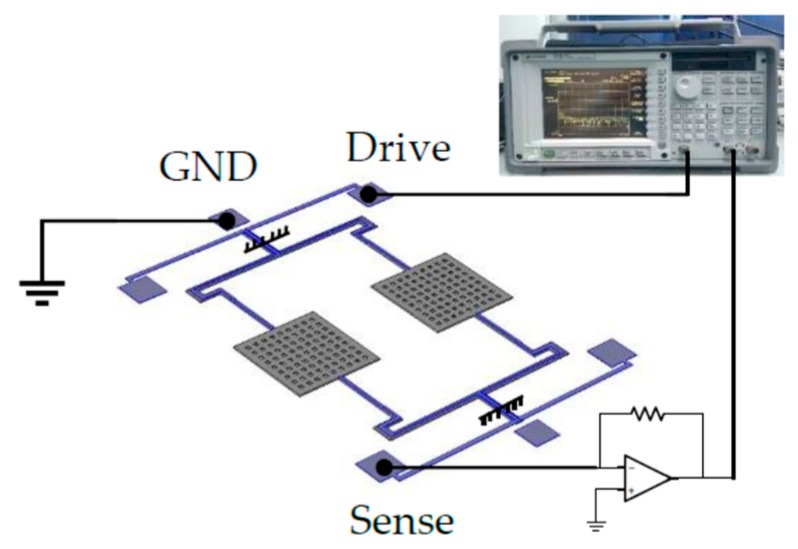
The schematics of the electrical test.

**Figure 8:**
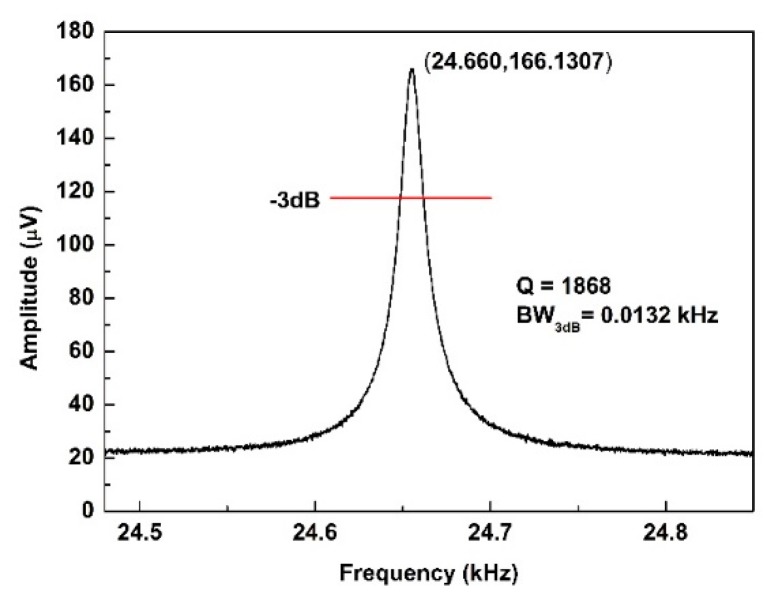
The resonant characteristics of the AlN accelerometer at a static state.

The characteristics of this AlN accelerometer were tested by the dynamic signal analyzer 35,670 A. The source signal, which comes from the 35,670 A, applies on the drive electrode to excite the structure. The amplitude of the input signal is 0.2 Vpk. The GND electrodes are the bottom electrode. It connects the ground. In addition, the resonant signal is detected from the sense electrode. Figure 7 shows the schematics of an electrical test of this accelerometer. The resonance frequency is 24.66 kHz at a static state at room temperature. The quality factor is 1868 as shown in Figure 8. 

**Figure 9:**
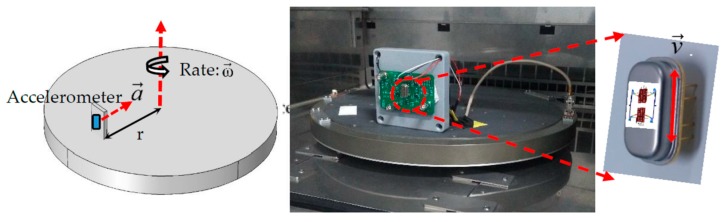
The sensitivity test by the rotating platform.

For the sensitivity test, the accelerometer was placed on the rotating platform vertically, as shown in Figure 9. A centripetal acceleration, which comes from the circular motion (i.e., $a=\omega^{2}r$), would apply on the AlN resonant accelerometer, and lead to a frequency shift. The rates of the rotating platform range from 0~1000°/s. The accelerometer is placed at the position 20.5 cm away from the center. In this test, the rate was set from zero to 1000°/s with a step of 100°/s. The room temperature is 25 °C.

**Figure 10:**
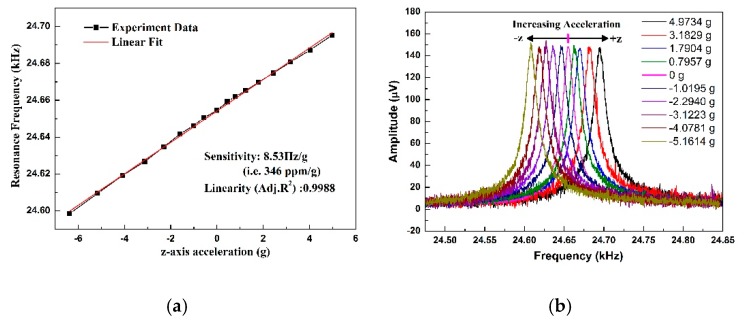
(**a**) Sensitivity of the accelerometer; (**b**) resonant peaks at different accelerations.

In the rotation test, the centripetal acceleration is perpendicular to the plane of the accelerometer. To avoid the Coriolis force, the MEMS chip is placed on the support vertically, which the direction of the vibration velocity $\vec{v}$ is parallel with the rate $\vec{\omega }$. Therefore, the Coriolis force is null. Figure 10 shows the experiment results of the relationship between the accelerations and resonance frequencies. The sensitivity of the AlN resonant accelerometer is 8.53 Hz/g, tested from −5 g to +5 g. The relative sensitivity (i.e., $\frac{\Delta f}{f_{0}}/g$, where *f_0_* is the resonance frequency) is 346 ppm/g at the base frequency of 24.66 kHz. The linearity of the accelerometer is 0.9988. With the increase of the acceleration, the resonance frequency increases. As shown in Figure 10b, the amplitude of each resonant peak is nearly equivalent.

**Figure 11:**
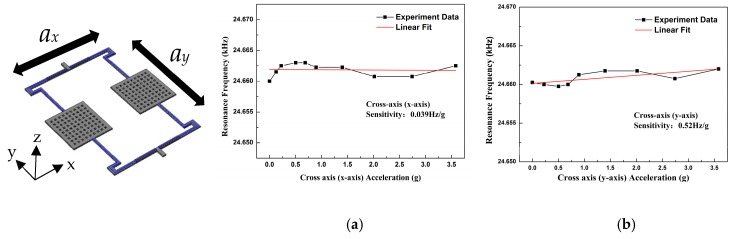
Cross axis sensitivities of the AlN MEMS accelerometer. (**a**) X-axis sensitivity; (**b**) Y-axis sensitivity.

The cross error of this MEMS accelerometer was characterized by the rotation test. Figure 11a,b show the test results. The output frequencies scatter around 24.66 kHz. The x-axis sensitivity is 0.039 Hz/g, and the y-axis sensitivity is 0.52 Hz/g. Compared with the z-axis sensitivity, the cross error of the x-axis is 0.46% and the y-axis is 6.1%. These results prove that this MEMS accelerometer is insensitive to the in-plane accelerations. 

**Figure 12:**
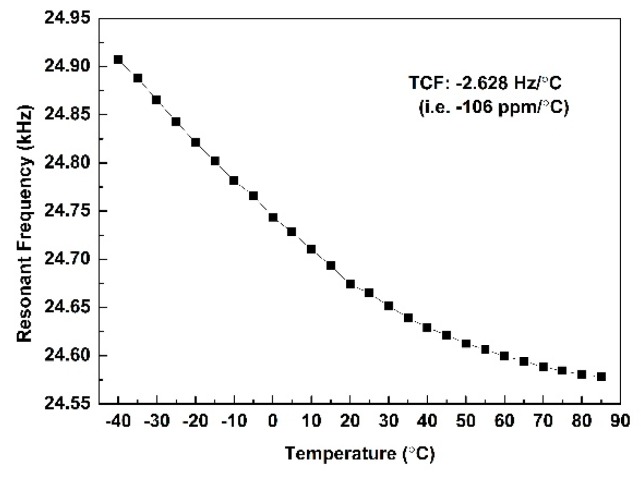
The temperature characteristic of the AlN accelerometer tested from −40 °C to 85 °C.

The temperature characteristic of the AlN accelerometer is tested. The temperatures range from −40 °C to 85 °C with a step of 5 °C. The temperature coefficient of frequency (TCF) of this accelerometer is −2.628 Hz/°C (i.e., −106 ppm/°C). Figure 12 shows the temperature characteristic of this accelerometer. Due to the negative temperature coefficient of AlN, a higher temperature will lead to a lower Young’s modulus. The resonance frequency is proportional to $\sqrt{\frac{E}{\rho }}$. Where *E* is the Young’s modulus. And *ρ* is the density.

(3) $$f_{0}\propto \sqrt{\frac{E}{\rho }}$$

Therefore, the resonance frequency will decrease with the increase of the temperature. In order to reduce the TCF, an optimized temperature compensation layer (SiO_2_ layer) or a temperature compensation circuit will be used. 

## 5. Conclusions

This paper reports an optimized AlN resonant accelerometer with a tuning-fork structure. The accelerometer is excited to vibrate in-plane. A z-axis acceleration, which is vertical to the plane of the accelerometer, can be detected. The z-axis sensitivity is 346 ppm/g, which is five times as much as the T-shape accelerometer. This accelerometer is potential to realize an integrated 3D AlN accelerometer. The Q value is 1868. The temperature coefficient of frequency (TCF) of this accelerometer is −106 ppm/°C, tested from −40 °C to 85 °C. A differential structure and a temperature compensation circuit will be designed to reduce the TCF. In the future work, we will focus on the increase of sensitivity, 3D integration, and reduction of TCF. 

## Figures and Tables

**Table 1:** The structure parameters of the AlN MEMS accelerometer.

Parameters	Dimensions
Length of the beam (AB)	1150 μm
Width of the beam (AB)	35 μm
Length of the beam (CD)	400 μm
Width of the beam (CD)	25 μm
Width of the electrodes	12 μm
The sides of the proof mass	500 μm
The sides of the releasing hole	20 μm
The thickness of the structure	2.05 μm

**Table 2:** The parameters of each material from COMSOL Multiphysics library.

Parameters	AlN	Mo	SiO_2_	Si	Pt	Ti
Density (kg/m^3^)	3300	10,200	2200	2329	21,450	4506
Young’s Modulus (GPa)	410	312	70	170	168	115
